# Effects of Boron Nutrition and Water Stress on Nitrogen Fixation, Seed *δ* ^15^N and *δ* ^13^C Dynamics, and Seed Composition in Soybean Cultivars Differing in Maturities

**DOI:** 10.1155/2015/407872

**Published:** 2015-01-18

**Authors:** Nacer Bellaloui, Alemu Mengistu

**Affiliations:** ^1^Crop Genetics Research Unit, USDA-ARS, Stoneville, MS 38776, USA; ^2^Crop Genetics Research Unit, USDA-ARS, Jackson, TN 38301, USA

## Abstract

Therefore, the objective of the current research was to investigate the effects of foliar B nutrition on seed protein, oil, fatty acids, and sugars under water stress conditions. A repeated greenhouse experiment was conducted using different maturity group (MG) cultivars. Plants were well-watered with no foliar B (W − B), well-watered with foliar B (W + B), water-stressed with no foliar B (WS − B), and water-stressed with foliar B (WS + B). Foliar B was applied at rate of 0.45 kg*·*ha^−1^ and was applied twice at flowering and at seed-fill stages. The results showed that seed protein, sucrose, fructose, and glucose were higher in W + B treatment than in W − B, WS + B, and WS − B. The increase in protein in W + B resulted in lower seed oil, and the increase of oleic in WS − B or WS + B resulted in lower linolenic acid. Foliar B resulted in higher nitrogen fixation and water stress resulted in seed *δ* ^15^N and *δ* ^13^C alteration. Increased stachyose indicated possible physiological and metabolic changes in carbon and nitrogen pathways and their sources under water stress. This research is beneficial to growers for fertilizer management and seed quality and to breeders to use ^15^N/^14^N and ^13^C/^12^C ratios and stachyose to select for drought tolerance soybean.

## 1. Introduction

Soybean is a major crop in the world, and its nutritional benefits reside in its seed protein (37–42%), oil (19–23%), fatty acids (palmitic 10–13%; stearic, 2–4%; oleic, 20–23%; linoleic, 52–59%; linolenic, 6–9%), and minerals contents [1–4]. Seeds with higher protein content are desirable for soymeal for livestock, and higher oleic acid and lower linolenic acid are desirable for oil oxidative stability and long shelf life of the oil. Mono- and disaccharides are desirable for taste, but high stachyose is undesirable because of its contribution to flatulence or diarrhea in nonruminants such as chicken and pig [5]. Therefore, increasing seed quality by targeting these desirable traits is critical for human nutrition health and livestock production.

It is well-known that seed composition (seed protein, oil, fatty acids, and sugars) is genetically controlled, but it is also reported that these constituents have been found to vary depending on biotic and abiotic stress factors, including water stress/drought, temperature, agriculture practices, fertilizer application, genotype, and maturity. For example, the influence of environment and maturity group on seed protein, oil, fatty acids, sugars, and minerals has been reported previously [4, 6–11]. In a multiyear experiment, it was investigated that the interaction between maturity and environment in six maturities (IIIII, IV, V, VI, VII, and VIII-IX) in 14 to 24 environments in each year for protein and oil, and found consistency of maturity group (MG) effect and its interaction with the environment (E) on protein and oil in 3-year multilocation soybean trials [9]. They found that the environment was the most important source of variation, except for 1 year for protein and oil content. However, the main effect of MG was greater than the effect of MG × E interaction for oil content and oil plus protein content. It was found that all environments produced high oil in cultivars belonging to MGs II-III and IV, but for protein MG × E interaction occurred in two MG × E combinations that produced the highest protein [9]. This means that, in some environments, MG VI protein was had the highest protein and in others MG II-III produced more protein. This trend was explained as consequences of high temperature during seed-fill that led a similar performance of MG and consistent pattern of higher oil content across seasons and environments in earlier MGs.

Boron nutrient is essential for crop growth, development, production, and seed quality [12–15]. Boron deficiency in soil due to biotic or abiotic stress factors results in yield loss and poor seed quality. Boron was reported to mainly have a structural involvement [16, 17], but metabolic involvement of B was also indicated [12, 13, 16]. Example, B is involved in nitrogen fixation [18], nodules [19, 20], nodulin protein (ENOD2) in nodule parenchyma cells and malfunction of oxygen diffusion barrier [21], B in carbohydrates metabolism [13], especially with sugar alcohols [14, 22], phenolic metabolism [13], ion uptake [13], plasma membrane-bound H+ATPase [23–25], and cell wall structure and membrane integrity [13, 26], seed protein, oil, fatty acids, and sugars [27]. Foliar B application to soybean has been previously reported [23, 28–31], but there is no clear evidence that B directly affects nitrogen metabolism [13, 21, 32] or seed protein, oil, fatty acids, and sugars [27].

Based on the above introduction it is clear that in spite of the well-established literature on the structural role [12, 13, 16] and metabolic role of B [18–21], information about foliar B application effects on seed composition (protein, oil, fatty acids, and sugars) is limited, especially under water stress conditions [27]. Therefore, the objective of the current research was to investigate the effects of foliar B application on seed protein, oil, fatty acids, and sugars. Since seed protein and oil production are associated with nitrogen and carbon fixation rates, dynamics of nitrogen fixation and natural abundance of *δ* ^15^N and *δ* ^13^C isotopes were also investigated.

## 2. Materials and Methods

### 2.1. Growth Conditions

The experiment was conducted under greenhouse conditions twice. Soybean seeds were germinated in flat trays in vermiculite, and uniform size seedlings at about V1 stage were transplanted into 9.45 L size pots filled with field soil. Physical and chemical analysis of soil showed that the soil was a Dundee silt loam (fine-silty, mixed, active, and thermic Typic Endoaquolls) with pH 6.3 and 1.1% organic matter. The soil contained enough B concentration (B concentration was 0.72 mg*·*kg^−1^). Water stress conditions were achieved by weighing soil in pots then saturating them with deionized water and were left to drain and weighed again to obtain the water field capacity [27]. Soil water potential sensors equipped with Soil Moisture Meter (WaterMark Company, Inc., WI, USA) were used for measurements. Treatments were well-watered plants with no foliar B (W − B), well-watered plants with foliar B (W + B), water-stressed plants with no foliar B (WS − B), and water-stressed plants with foliar B (WS + B). Water stressed plants were kept between –90 and –100 kPa and well-watered plants were kept between –15 and –20 kPa (this was considered field capacity for the control plants). Foliar B was applied at rate of 0.45 kg*·*ha^−1^ and was applied twice at flowering and at seed-fill stages. Leaf samples for B measurements were taken after the second application at seed-fill stage. Seeds at harvest maturity were collected for seed nutrition assessments. Four replicates were used for each treatment and each pot with four individual plants was considered one replicate. The greenhouse conditions were as follows: temperature ranged from about 32°C ± 11°C during the day and about 29°C ± 9°C at night with a photosynthetic photon flux density (PPFD) of about 1800–2500 *μ*mol*·*m^−2^ *·*s^−1^, as measured by Quantum Meter (Spectrum Technology, Inc., IL, USA). The two experiments were conducted simultaneously at the same time and during the normal growing season (April-September) for the Early Soybean Production System in the midsouth USA.

### 2.2. Boron Determination

Boron concentrations in leaves and seeds were determined using the Azomethine-H method [15, 33]. Briefly, samples of 1.0 g were ashed at 500°C, extracted with 20 mL of 2 M HCl at 90°C for 10 minutes, and filtered, and then a 2 mL sample of the filtered mixture was added to 4 mL of buffer solution (containing 25% ammonium acetate, 1.5% EDTA, and 12.5% acetic acid). A volume of 4 mL of freshly prepared azomethine-H solution (0.45% azomethine-H and 1% of ascorbic acid) was added. The concentrations of B in leaves and seeds were determined in the samples at 420 nm using a Beckman Coulter DU 800 spectrophotometer (Beckman Coulter, Inc., Brea, CA, USA) [34]. Soil boron was analyzed at The University of Georgia's Soil, Plant, and Water Laboratory, Athens, GA. The concentration of B was determined using a 5 g soil : 20 mL Mehlich-1 solutions and analyzed using inductively coupled plasma (ICP) spectrometry.

### 2.3. Seed Analysis for Protein, Oil, and Fatty Acids

Seeds at harvest maturity were collected from each treatment and analyzed for protein, oil, and fatty acids. Briefly, a sample of 25 g of seed was ground using the Laboratory Mill 3600 and analyzed by near infrared reflectance [10, 35] using a diode array feed analyzer AD 7200 (Perten, Springfield, IL, USA). A calibration equation was developed by the University of Minnesota using Perten's Thermo Galactic Grams PLS IQ software, and the calibration curve was established using AOAC methods [36, 37]. Protein and oil contents were determined based on a seed dry weight basis [27, 35, 38], and concentrations of palmitic, stearic, oleic, linoleic, and linolenic fatty acids were conducted on the total oil basis [27].

### 2.4. Seed Analysis for Sucrose, Raffinose, and Stachyose

Seeds at harvest maturity were collected and analyzed for sugars. Briefly, a sample of 25 g of seed from each treatment was ground using the Laboratory Mill 3600 and analyzed by near infrared reflectance (NIR) [11, 34, 35] using the AD 7200 array feed analyzer. Analyses of sugars were performed based on a seed dry weight basis [34, 35, 38].

### 2.5. Glucose Determination in Seed

Glucose concentration in seed was conducted by an enzymatic reaction using a Glucose (HK) Assay Kit, Product Code GAHK-20 (Sigma-Aldrich Co., St. Louis, MO, USA) [27]. In this reaction, glucose is phosphorylated by adenosine triphosphate (ATP) and catalyzed by hexokinase. Then, the glucose-6-phosphate (G6P) produced is oxidized to 6-phosphogluconate by oxidized nicotinamide adenine dinucleotide (NAD) in a reaction catalyzed by glucose-6-phosphate dehydrogenase (G6PDH). An equimolar amount of NAD is then reduced to NADH, and the increase in absorbance at 340 nm is directly proportional to the glucose concentration in the sample. The procedure was that seed samples were ground using the Laboratory Mill 3600, and a random sample of 0.1 mg was extracted with deionized water, and the sample solution was heated using heat plate to aid extraction. Then, the extract was diluted to 1 : 100 with deionized water to obtain a range of 0.05 to 5 mg glucose mL^−1^. A volume of 100 *μ*L sample was added to 1 mL of the Glucose (HK) Assay Reagent and incubated at room temperature for 15 min. Glucose concentration in samples was determined at absorbance of 340 nm using the Beckman Coulter DU 800 spectrophotometer. The concentration of glucose was expressed as mg g dwt^−1^.

### 2.6. Fructose Determination in Seed

Fructose concentration was determined by an enzymatic reaction using a Fructose Assay Kit, Product Code FA-20 (Sigma-Aldrich Co., St. Louis, MO, USA) [27]. In this reaction, fructose is phosphorylated by ATP in a reaction catalyzed by hexokinase. Fructose 6-phosphate is then converted to G6P by phosphoglucose isomerase (PGI), and G6P then was oxidized to 6-phosphogluconate in the presence of NAD in a reaction catalyzed by glucose-6-phosphate dehydrogenase (G6PDH). The increase in absorbance at 340 nm is directly proportional to fructose concentration in a sample. The procedure method was that seed samples were ground using the Laboratory Mill 3600 and extracted as described above in glucose determination. Fructose concentration was determined by reading samples at 340 nm using the Beckman Coulter DU 800 spectrophotometer. Fructose concentration of seeds was expressed as mg g dwt^−1^.

### 2.7. Analysis of *δ* ^15^N (^15^N/^14^N Ratio) and *δ* ^13^C (^13^C/^12^C Ratio) Using Natural Abundance

Analysis of *δ* ^15^N and ^13^C natural abundance was conducted from ratios of nitrogen isotope (^15^N/^14^N ratio) and carbon isotope (^13^C/^12^C ratio) using about 0.9 mg of ground seeds. Isotopic analysis was conducted using a Thermo FinniGlyn Delta Plus Advantage Mass Spectrometer with a FinniGan ConFlo III, and Isomass Elemental Analyzer (Bremen, Germany). Isodat software version 2.38 was used to obtain Delta values [39–41]. The elemental combustion system was Costech ECS 4010 with an autosampler (Bremen, Germany).

### 2.8. Experimental Design and Statistical Analysis

The experiment was a split plot design with irrigation as a main plot, cultivar, and subplot, and B treatments were sub-subplot. The data were subjected to analysis of variance using Proc ANOVA in SAS [42]. Means were separated by Fisher's least significant difference test at the 5% level of probability. Four replicates from each treatment were used. Data were combined and pooled across experiments because there were no interactions between experiments and other source effect factors.

## 3. Results and Discussion

Analysis of variance (ANOVA) showed that cultivar (Cv), water treatment (W, either well-watered or water-stressed plants), and boron treatment (Treat) had significant effects on protein, oil, oleic, linoleic, and linolenic acids (Table 1). Generally, there were no interactions between experiments (E) and other factors, indicating that the treatments (W, Treat, Cv) had the same effect in each experiment. Therefore, data were combined and pooled across the two experiments. The interaction between Treat, Cv, and W indicated that the B treatments were dependent on the cultivar and whether the plants were stressed or not. There were no significant effects of the studied factors on palmitic and stearic acids. Similar effects were shown for nitrogen fixation (ARA) and sugars, except for raffinose and stachyose, which were not affected by Treat, W, and Cv (Table 2).

### 3.1. Effects of Foliar B Application and Water Stress on Protein, Oil, and Fatty Acids

Under well-watered conditions (Table 3), foliar boron (FB) resulted in higher seed protein in all cultivars, higher oleic acid in MG V cultivars only, and higher linolenic acid in MG III cultivars only. An inverse relationship between protein and oil and between oleic and linolenic acids was noticed in each cultivar. Both palmitic and stearic acids were not responsive to FB application. Under water stress conditions (Table 3), FB application resulted in higher concentrations of protein and oleic acid. No consistent effects of FB on linolenic acid and oil concentrations and no significant effects of FB on palmitic and stearic acids were observed. Cultivars accumulated different concentration of seed constituent components, and MG V tended to accumulate more protein than MG III cultivars, but MG III accumulated higher concentrations of oil than in MG V. Under water stress conditions, plants accumulated higher protein and oleic acid and lower linolenic acid than under well-watered conditions. The higher protein and oleic acid concentrations with FB application indicated the positive effects of FB on protein and fatty acid production under adequate soil moisture conditions. The mechanism of how B effects seed protein and oleic acid accumulation is not well known [27] but could be due to its positive indirect effects on nitrogen and carbon fixation rates. Previous research showed that levels of B in soil and leaves were associated with seed protein and oleic fatty acid [10, 27] and FB application resulted in higher protein and oleic acid [3, 27]. The inverse relationships between protein and oil [43] or between oleic and linolenic acid [34] were previously reported. The increase of protein and oleic acid concentrations under water stress was due to small seed size and seed weight. Seed weight of plants under water stress condition was lower than in seed of well-watered plants (data not shown). Therefore, the causative factor of the increase of protein and oleic acid under well-watered conditions is different than that of the increase of protein and oleic acid under water stress concentrations. The higher concentrations of protein in MG V cultivars could be due to a longer maturity period in MG V cultivars resulting in longer period of protein accumulation in seeds compared with MG III. In spite of the inconsistent results reported in the literature regarding the effects of FB on seed composition, our current research showed that FB altered seed composition. Further research is needed to understand the mechanisms on how these effects occur.

### 3.2. Effects of Foliar B Application and Water Stress on Seed Sugars

In well-watered plants (Table 4), FB application resulted in higher sucrose, glucose, and fructose in both MG III and V cultivars. No consistent effects were observed in raffinose and stachyose in all cultivars, although MG V cultivars accumulated higher stachyose concentrations. Similar observation was recorded in water-stressed plants (Table 4), except for Pella 86 where fructose did not show significant effects by FB may be due to cultivar differences. Also, it appears that plants under water stress accumulated less sugars than in well-watered conditions, except for stachyose. Effects of B on sugars were previously reported, but the literature was related to sugars translocation and sugar metabolism [13, 44]. The higher accumulation of sucrose, glucose, and fructose, resulted from FB, indicated that B may have indirect stimulating effects on these sugars translocation to seeds or due to B positive effects on nitrogen and carbon fixation rates in well-watered plants. The lower accumulation of sucrose, glucose, and fructose under water stress conditions indicated the sensitivity of these sugars to water stress due to either lower sugar movement to seed or a decrease of photoassimilates due to lower nitrogen and carbon fixation under water stress. Our current results on nitrogen fixation and ^15^N and ^13^C isotopes, below, indicated lower nitrogen fixation rates and alteration in ^15^N and ^13^C under water stress conditions, indicating changes in nitrogen and carbon fixation pathways. On the other hand, nitrogen fixation rates were higher and no alteration in ^15^N and ^13^C isotopes occurred in well-watered conditions.

Further, it was found that the activity of sucrose synthase, the main enzyme involved in sucrose hydrolysis in nodules, was significantly inhibited under drought conditions [45, 46], which may indicate that supply of sucrose, glucose, and fructose is more sensitive to water stress than that of raffinose and stachyose. Boron involvement in sugar metabolism could be due to the high permeability of boron across membranes, and foliar boron can enter the phloem and form a complex with sugars and retranslocate to the inflorescence, impacting sugar metabolism [13].

The higher accumulation of stachyose concentration under water stress may indicate possible role of this oligosaccharide in abiotic stress conditions. Effects of water stress on sugars were previously reported [27, 45, 47]. For example, it was reported that raffinose and galactinol levels may play an important role in plant tolerance to biotic and abiotic stress [27], and the accumulation of galactinol and raffinose may have a protective role in plants from stress environment, especially drought [27]. The biological functions of raffinose and stachyose are still not well known [48], although oligosaccharides (sucrose, raffinose, and stachyose) are related to seed quality [49] and desiccation tolerance during seed maturation and protection of seeds against damage during seed dehydration. Previous research showed that the accumulation of compatible solutes such as sugars may protect plants against stress environments [50], and nonstructural carbohydrates (sucrose, hexoses, and sugar alcohols) were found to have a strong correlation between sugar accumulation and osmotic stress tolerance [47]. It was suggested that sugars act as osmotica and contribute to the stabilization of membrane structures, protecting cells during desiccation [51], and interact with polar headgroups of phospholipids in cell membranes to prevent membrane fusion. Our experiment showed higher seed stachyose accumulation under water stress, reflecting possible role in drought stress, supporting previous findings [47]. It is well known that soybean sugars contribute to seed quality; that is, seed with high raffinose and stachyose concentrations are undesirable because they have negative effects on the nutritive value of soymeal and are indigestible by humans and monogastric animals, causing flatulence or diarrhea [7]. On the other hand, high level of seed sucrose, glucose, and fructose is desirable because it improves taste and flavor of tofu, soymilk, and natto [2]. In spite of this, mechanisms of how these compounds are involved in stress tolerance are still not fully understood [50, 52], and the relationship between sucrose, raffinose, and stachyose is still not well established, although it was found to be affected by genotype and environment and their interactions.

### 3.3. Dynamics of *δ* ^15^N (^15^N/^14^N Ratio) and *δ* ^13^C (^13^C/^12^C Ratio) Natural Abundance

**Figure 1:**
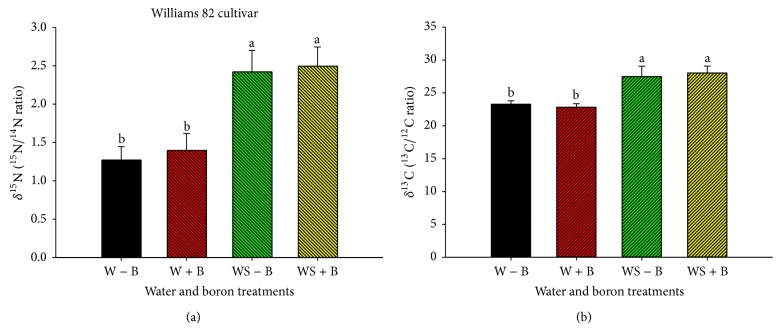
*δ* ^15^N (^15^N/^14^N ratio) (a) and *δ* ^13^C (^13^C/^12^C ratio) (b) natural abundance values as changed by water stress and boron treatments (foliar boron was applied at 0.45 kg·ha^−1^ at flowering and seed-fill stages) in Williams 82 cultivar (maturity group III). Treatments were as follows: well-watered plants with no foliar B (W − B), well-watered plants with foliar B (W + B), water-stressed plants with no foliar B (WS − B), and water-stressed plants with foliar B (WS + B).

**Figure 2:**
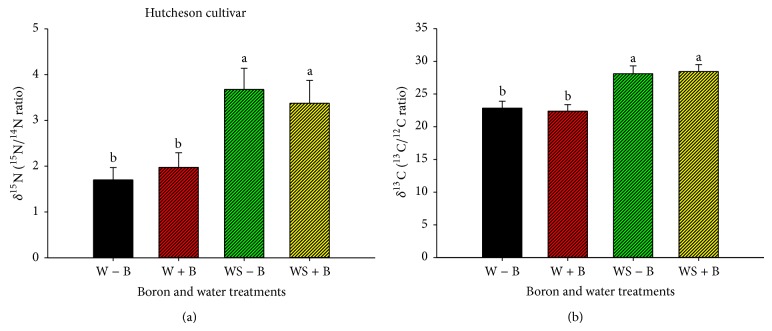
*δ* ^15^N (^15^N/^14^N ratio) (a) and *δ* ^13^C (^13^C/^12^C ratio) (b) natural abundance values as changed by water stress and boron treatments (foliar boron was applied at 0.45 kg·ha^−1^ at flowering and seed-fill stages) in Hutcheson cultivar (maturity group V). Treatments were as follows: well-watered plants with no foliar B (W − B), well-watered plants with foliar B (W + B), water-stressed plants with no foliar B (WS − B), and water-stressed plants with foliar B (WS + B).

Application of FB did not alter ^15^N/^14^N and ^13^C/^12^C ratios (Figures 1(a), 1(b), 2(a), and 2(b)). However, water stress resulted in alteration of ^15^N/^14^N and ^13^C/^12^C ratios in both Williams 82 and Hutcheson. The alteration in ^15^N/^14^N is indicated by increasing ^15^N (derived from soil nitrogen that is used for nitrate assimilation) and decreasing ^14^N (derived from atmospheric nitrogen, which is used for nitrogen fixation). Also, the alteration of ^15^N/^14^N indicated that water stress inhibited nitrogen fixation due to nitrogenase sensitivity to water stress (Table 4). This shift may indicate a possible mechanism where soil nitrogen (source of nitrogen assimilation) was used to compensate for the inhibition of nitrogen fixation under water stress (Table 4). The increase of ^13^C/^12^C ratio indicated an enrichment of ^13^C and alteration of carbon source.

The possible mechanisms of how plants shift *δ* ^15^N to compensate for the inhibition of nitrogen fixation under water stress are not well understood. Previous research reported that the *δ* ^15^N values in the xylem and plant tissues were related to the acquired N, and the *δ* ^15^N value can be altered due to N metabolism [39, 40, 53]. The higher enrichment of *δ* ^13^C (higher ^13^C/^12^C ratio) in seed of plants grown under water stress conditions also indicated change in carbon fixation source. Previous research reported that *δ* ^13^C value in plant tissues can be affected by water supply and temperature [54], plant physiology [55], and mycorrhizal infection [56]. This indicated that the abundance of *δ* ^13^C in plant tissues is affected by environmental conditions (biotic or abiotic factors, including drought), and this occurs by affecting plant gas exchange through stomatal conductance and CO_2_ fixation [57, 58]. The shift in ^13^C/^12^C ratio indicates that drought stress led to stomatal closure and ^13^C fixation increase, leading to less discrimination against *δ* ^13^C and a shift in carbon fixation metabolism from ribulose bisphosphate (RuBP) carboxylase pathway to phosphoenolpyruvate carboxylase (PEP), resulting in *δ* ^13^C enrichment [54]. Our current research demonstrated that *δ* ^15^N and *δ* ^13^C values changed and water stress resulted in enrichment of *δ* ^15^N and *δ* ^13^C. This indicated that nitrogen and carbon metabolism pathways altered, explaining the possible association between nitrogen and carbon fixation pathways and seed protein, oil, and sugars accumulation in seed, impacting seed quality.

**Figure 3:**
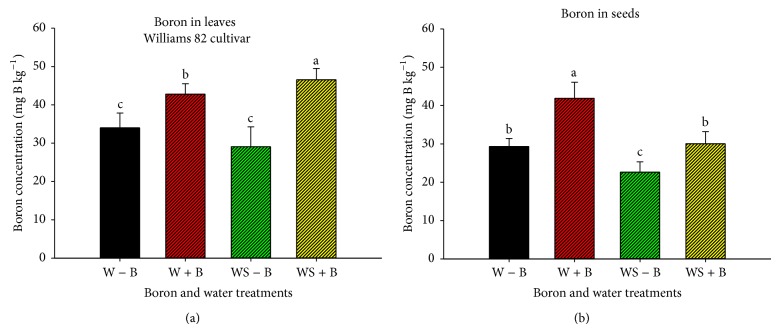
Concentration (mg B kg^−1^) of boron in leaves (a) and seed (b) as influenced by water stress and boron treatments (foliar boron was applied at 0.45 kg·ha^−1^ at flowering and seed-fill stages) in Williams 82 cultivar (maturity group III). Treatments were as follows: well-watered plants with no foliar B (W − B), well-watered plants with foliar B (W + B), water-stressed plants with no foliar B (WS − B), and water-stressed plants with foliar B (WS + B).

**Figure 4:**
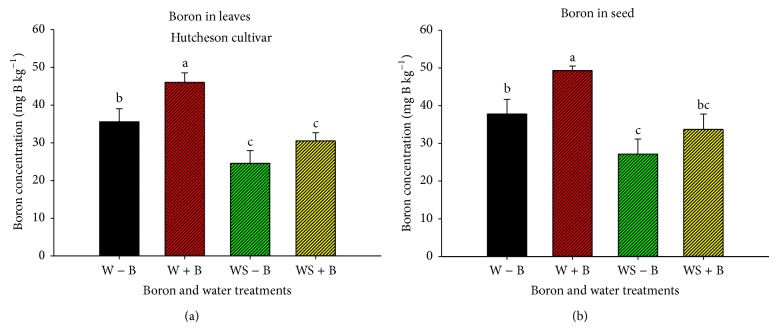
Concentration (mg B kg^−1^) of boron in leaves (a) and seed (b) as influenced by water stress and boron treatments (foliar boron was applied at 0.45 kg·ha^−1^ at flowering and seed-fill stages) in Hutcheson cultivar (maturity group V). Treatments were as follows: well-watered plants with no foliar B (W − B), well-watered plants with foliar B (W + B), water-stressed plants with no foliar B (WS − B), and water-stressed plants with foliar B (WS + B).

Foliar B application (Figures 3 and 4) increased leaf and seed B in well-watered and water-stressed plants in all cultivars, although the accumulation of B in leaves and seed was different in each cultivar, possibly due to cultivar/genotype differences and maturity. Boron concentration was higher in leaves than in seeds under water stress conditions, which may be due to limited translocation of B from leaves to seed, indicating that FB application under drought stress may amplify B deficiencies during grain-fill stage. Also, the lower concentration of B in seeds of plants grown under water stress could be due to limited translocation of B from leaves to seed.

## 4. Conclusions

The current research demonstrated that foliar B application and water stress altered seed composition, especially protein, oleic acid, and sugars. Foliar B application did not alter the dynamics of *δ* ^15^N (^15^N/^14^N ratio) and *δ* ^13^C (^13^C/^12^C ratio) isotopes, but water stress resulted in shifting nitrogen metabolism towards nitrogen assimilation due to higher sensitivity of nitrogen fixation to water deficit. The lower accumulation of B in seeds in water-stressed seeds may be due to limited translocation of B from leaves to seed.

## Figures and Tables

**Table 1:** Analysis of variance for the effect of experiment (E), cultivar (Cv), water treatment (W: well-watered plants and water-stressed plants), boron treatments (foliar boron was applied at 0.45 kg·ha^−1^ at flowering and seed-fill stages), and their interactions on seed protein, oil, fatty acids (g kg^−1^), and boron (mg B kg^−1^).

Source effect	Protein	Oil	Palmitic	Stearic	Oleic	Linoleic	Linolenic	Boron
Experiment (E)	NS	NS	NS	NS	NS	NS	NS	NS
Cultivar (CV)	∗∗∗	∗∗∗	NS	∗	∗∗	∗	∗	∗∗
Water treatment (W)	∗∗∗	∗∗	NS	NS	∗∗	∗	∗∗∗	∗∗∗
Treatment (Treat)	∗∗∗	∗∗	NS	NS	∗∗	∗	∗∗	∗∗∗
E × CV	NS	NS	NS	NS	NS	NS	NS	NS
E × W	NS	NS	NS	NS	NS	NS	NS	NS
E × Treat	NS	NS	NS	NS	NS	NS	NS	NS
CV × W	∗∗	∗	NS	NS	∗∗	∗	∗∗	∗∗
CV × Treat	∗	∗	NS	∗	∗∗∗	∗	∗∗	∗
W × Treat	∗	∗	NS	NS	∗∗	∗	∗∗∗	∗∗
E × CV × W × Treat	∗	∗	NS	NS	∗	∗	∗	∗

∗: significance at *P* ≤ 0.05; ∗∗: significance at *P* ≤ 0.01; ∗∗∗: significance at *P* ≤ 0.001.

**Table 2:** Analysis of variance for the effect of experiment (E), cultivar (Cv), water treatment (W: well-watered plants and water-stressed plants), boron treatments (foliar boron was applied at 0.45 kg·ha^−1^ at flowering and seed-fill stages), and their interactions on seed sugars (mg g^−1^), nitrogen fixation rate (ARA), and natural abundance of nitrogen and carbon isotopes (^15^N/^14^N ratio; ^13^C/^12^C ratio).

Source effect	Sucrose	Raffinose	Stachyose	Glucose	Fructose	ARA	^ 15^N/^14^N	^ 13^C/^12^C
Experiment (E)	NS	NS	NS	NS	NS	NS	NS	NS
Cultivar (CV)	∗∗	∗∗	∗∗	∗∗∗	∗∗	∗∗	NS	NS
Water treatment (W)	∗	NS	∗∗∗	∗∗	∗∗	∗∗∗	∗∗	∗∗
Treatment (Treat)	∗∗	∗∗	∗∗∗	∗	∗	∗∗	NS	NS
E × CV	NS	NS	NS	NS	NS	NS	NS	NS
E × W	NS	NS	NS	NS	NS	NS	NS	NS
E × Treat	NS	NS	NS	NS	NS	NS	NS	NS
CV × W	∗	NS	∗∗	∗∗	∗	∗∗	∗	∗
CV × Treat	NS	NS	NS	NS	NS	NS	NS	NS
W × Treat	∗	∗	∗∗	∗	∗	∗	NS	NS
E × CV × W × Treat	∗	NS	∗	∗	∗	∗	NS	NS

∗: significance at *P* ≤ 0.05; ∗∗: significance at *P* ≤ 0.01; ∗∗∗: significance at *P* ≤ 0.001.

**Table 3:** Effects of water stress on seed protein, oil, and fatty acids (g kg^−1^) in maturity group (MG) III (Williams 82 and Pella 86) and MG V (Hutcheson and Freedom) soybean cultivars. Plants were grown under well-watered and water-stressed conditions and boron was foliar applied at 0.45 kg·ha^−1^ at flowering and seed-fill stages.

Genotype	Well-watered plants (soil water potential = −15 to −20 kPa)							
	B treatment	Protein	Oil	Palmitic (C16:0)	Stearic (C18:0)	Oleic (C18:1)	Linoleic (C18:2)	Linolenic (C18:3)
Williams 82 (MG III)	−B	404 e	222 a	112 a	32.3 a	255 a	523 b	67.4 d
	+B	412 d	225 a	113 a	25.6 a	245 a	532 b	65.3 d
Pella 86 (MG III)	−B	417 d	216 b	114 a	24.4 a	243 a	542 b	65.7 d
	+B	431 b	215 b	113 a	24.5 a	253 a	537 b	63.4 d
Hutcheson (MG V)	−B	423 c	201 c	124 a	31.4 a	202 b	597 a	86.7 b
	+B	438 a	203 c	121 a	35.8 a	258 a	576 a	76.9 c
Freedom (MG V)	−B	435 b	205 c	113 a	32.3 a	217 b	586 a	97.2 a
	+B	444 a	210 c	115 a	33.8 a	264 a	568 a	75.5 c

Genotype	Water-stressed plants (soil water potential = −90 to −100 kPa)							
	B treatment	Protein	Oil	Palmitic (C16:0)	Stearic (C18:0)	Oleic (C18:1)	Linoleic (C18:2)	Linolenic (C18:3)

Williams 82 (MG III)	−B	422 e	214 a	117 a	24.1 a	276 b	543 a	54.7 b
	+B	436 d	215 a	124 a	26.4 a	333 a	534 b	65.3 a
Pella 86 (MG III)	−B	436 d	197 b	116 a	25.6 a	286 b	526 b	61.5 ab
	+B	441 c	201 b	122 a	32.3 a	325 a	536 b	67.7 a
Hutcheson (MG V)	−B	440 c	191 b	116 a	31.4 a	286 b	547 a	63.8 ab
	+B	453 b	186 c	118 a	27.7 a	326 a	532 b	62.5 ab
Freedom (MG V)	−B	446 c	175 c	119 a	29.4 a	266 b	552 a	64.8 a
	+B	469 a	18.5 c	117 a	31.7 a	336 a	547 a	57.4 b

Means within a column of each water treatment followed by the same letter are not significantly different at the 5% level as determined by Fishers' LSD test. Values are means of four replicates.

**Table 4:** Effects of water stress on seed sugars (mg g^−1^) and nitrogen fixation rate (ARA: [(*μ*mol C_2_H_2_ g nodule^−1^ h^−1^)] in maturity group (MG) III (Williams 82 and Pella 86) and MG V (Hutcheson and Freedom) soybean cultivars. Plants were grown under well-watered and water-stressed conditions and boron was foliar applied at 0.45 kg·ha^−1^ at flowering and seed-fill stages.

Genotype	Well-watered plants (soil water potential = −15 to −20 kPa)						
	B treatment	Sucrose (C_12_H_22_O_11_)	Raffinose (C_18_H_32_O_16_)	Stachyose (C_24_H_42_O_21_)	Glucose (C_6_H_12_O_6_)	Fructose (C_6_H_12_O_6_)	ARA (*μ*mol C_2_H_2_ g nodule^−1^ h^−1^)
Williams 82 (MG III)	−B	30.4 d	5.3 c	25.5 b	1.3 c	0.76 b	657 c
	+B	38.6 c	6.5 b	26.5 b	2.5 ab	0.99 a	878 a
Pella 86 (MG III)	−B	33.5 d	7.5 a	24.7 b	1.1 c	0.65 b	576 d
	+B	41.8 b	6.8 b	25.4 b	2.4 ab	0.92 a	745 b
Hutcheson (MG V)	−B	44.7 b	7.5 a	32.5 a	1.6 c	0.65 b	658 c
	+B	48.6 a	6.9 b	30.6 a	2.7 a	0.98 a	895 a
Freedom (MG V)	−B	38.6 c	7.4 a	31.5 a	2.3 b	0.76 b	758 b
	+B	53.6 a	8.0 a	33.6 a	2.8 a	1.23 a	976 a

Genotype	Water-stressed plants (soil water potential = −90 to −100 kPa)						
	B treatment	Sucrose (C_12_H_22_O_11_)	Raffinose (C_18_H_32_O_16_)	Stachyose (C_24_H_42_O_21_)	Glucose (C_6_H_12_O_6_)	Fructose (C_6_H_12_O_6_)	ARA (*μ*mol C_2_H_2_ g nodule^−1^ h^−1^)

Williams 82 (MG III)	−B	21.4 e	6.5 b	34.3 b	0.85 c	0.56 c	352 b
	+B	25.4 d	6.3 b	33.5 b	1.3 b	0.63 b	436 a
Pella 86 (MG III)	−B	20.5 e	6.6 b	32.5 b	0.75 c	0.40 d	267 d
	+B	33.2 b	7.5 a	33.8 b	1.3 b	0.56 d	315 c
Hutcheson (MG V)	−B	32.5 b	6.4 b	45.3 a	0.94 c	0.53 b	365 b
	+B	39.6 a	6.7 b	43.7 a	1.2 b	0.82 a	476 a
Freedom (MG V)	−B	28.6 c	7.1 a	45.7 a	1.4 b	0.63 b	326 b
	+B	35.7 b	6.5 b	42.8 a	2.1 a	1.10 a	476 a

Means within a column of each water treatment followed by the same letter are not significantly different at the 5% level as determined by Fishers' LSD test. Values are means of four replicates.

## Abbreviations

W − B: Well-watered plants, control, with no foliar boron
W + B: Well-watered plants with foliar boron
WS − B: Water-stressed plants with no foliar boron
WS + B: Water-stressed plants with foliar boron
B: Boron.
